# From apparent to true – from frequency to distributions (II)

**DOI:** 10.3325/cmj.2020.61.381

**Published:** 2020-08

**Authors:** Branimir K., Hackenberger

**Affiliations:** Department of Biology, Josip Juraj Strossmayer University, Osijek, Croatia *hackenberger@biologija.unios.hr*

The coronavirus disease 2019 (COVID-19) pandemic has compelled us to make decisions related to its curbing and management based on very limited data. Unfortunately, many countries do not have an elaborate decision-making system grounded in quantitative indicators and concrete research, but solely in personal opinions or political assessments. This has resulted in completely different approaches in individual countries and a relatively rapid switch from an epidemic into a pandemic. Furthermore, the quality of publicly available data largely differs regarding their origin and the time when they were obtained. The current statistics about this pandemic published around the world do not take into account two important things, their uncertainty and the possible causes of this uncertainty.

Due to the media hype and daily reporting, at first glance there seems to be a lot of publicly available data on the COVID-19 pandemic. Unfortunately, this is not the case. Almost all countries have serious data gaps, which prevent an unbiased and independent analysis of the actual prevalence and mortality rates. The impression of secrecy and non-transparency thus created fosters various conspiracy theories. It is therefore important to raise the quality level of publicly available data by using up-to-date and sophisticated methods of data mining and processing.

The Bayesian approach is generally recognized as more efficient and advanced compared with the commonly applied, frequentist, approach (1). During the COVID-19 pandemic, an increasing number of scientific articles that show the advantages of the Bayesian approach has been published. Some of these articles aim to model and forecast the spread of the pandemic, while others evaluate the effectiveness of individual therapies or assess the efficiency and reliability of diagnostic methods.

## Bayesian statistics in modeling the spread of COVID-19

According to Roda et al (2), one of the main reasons for the variability in predicting the COVID-19 epidemic is the lack of data on the actual dynamics of the infection spread, which results in so-called nonidentifiability in model calibration. Namely, it is possible to fit numerous models with different parameters to the same confirmed-case data with equal goodness of fit. The existence of nonidentifiability means that in the calibration step it is possible to determine several different combinations of model parameters (eg, case-infection ratio ρ, transmission rate b, etc) that will describe the input data equally well. Thus, the selection of some, equally likely, combination of parameters can result in significantly different prediction than other combinations of parameters. The way in which nonidentifiability is addressed in the model calibration process greatly affects the accuracy, reliability, and quality of model predictions. The authors used the SIR and SEIR frameworks to model the COVID-19 epidemic in Wuhan, which they calibrated using an improved model calibration method with Bayesian inference and affine invariant ensemble Markov chain Monte Carlo algorithm. This approach overcomes the problem of nonidentifiability by ensuring fast convergence to the target posterior distribution and provides more reliable credible intervals and model predictions. Arias Velásquez and Mejía Lara (3) used a similar approach to predict the course of the epidemic in the USA, but extended it in accordance with the observed unpredictability of the epidemic. Namely, the authors propose that the pandemic behaves as a dynamic-chaotic system, so they used a novel Bayesian method for chaotic dynamical systems, reduced-space Gaussian process regression. Moreover, De Simone and Piangerelli (4) presented a Bayesian procedure to quantify the impact of undetected infectious cases on the effective reproduction number, *R_t_*. The application of this framework on data regarding the number of confirmed cases in eight affected countries showed that the undetected cases increased the effective reproduction number by factors of order 2 to 10. By assuming that the spread of influenza and severe acute respiratory syndrome coronavirus 2 (SARS-CoV-2) was conditioned and limited by similar environmental and behavioral factors in early 2020, Du et al (5) estimated the early prevalence of symptomatic COVID-19 cases in Wuhan and Seattle based on the ratio of SARS-CoV-2 to influenza test positivity. The authors determined the model parameters using the Bayesian approach and Markov chain Monte Carlo, and concluded that the COVID-19 epidemics in Wuhan and Seattle had likely been spreading for several weeks before they became apparent and were far more extensive than initially reported. In addition to their applicability in country-level epidemiological modeling, Bayesian statistics has been used to estimate the parameters of models describing the total number of confirmed cases in the world. Maleki et al (6) used TP-SMN-AR models (autoregressive model based on the two-piece t distributions) and Bayesian information criteria as a model selection criterion.

Bayesian approaches have also been used to assess the effects of social distancing measures on the dynamics of the epidemic in several affected countries. Feroze (7) used Bayesian structural time series models to investigate the pattern of SARS-CoV-2 spread in India, Brazil, USA, Russia, and the UK between March 1 and June 29, 2020 to assess the impact of mitigation measures and predict the dynamics of the epidemic over the next 30 days. Compared with the usual ARIMA (auto regressive integrated moving average) time series model, the Bayesian inference-based approach produced more accurate forecasts, which is a prerequisite for timely action and planning of epidemic control strategies. Iwata et al (8) focused on the impact of school closures on mitigating the spread of the coronavirus in Japan. For this purpose, they performed a time series analysis with Bayesian statistics and found that school closures did not reduce the incidence of infection. Dehning et al (9) used the SIR epidemiological model framework in combination with Bayesian inference to analyze the effective growth rate of the number of new cases over time. They proved the applicability of their approach on data for Germany, where they found several points of change in the effective growth rate that correlated with the introduction of social distancing measures. Therefore, using a time-dependent spreading rate model, the authors quantified the effect of social distancing measures on the dynamics of the epidemic. The proposed model could also be used to precisely and realistically predict and simulate the future course of the epidemic.

Due to the variable and stochastic nature of the data on the number of confirmed cases, forecasting artificial intelligence models have also been used to predict the number of new cases. For example, da Silva et al (10) compared the predictive capacity of several artificial neural networks (ANNs). ANNs were trained on a time series of cumulative number of cases and climatological data (temperature, precipitation) for Brazil and the USA. Among the models examined, the Bayesian regression neural network ranked second for both states based on its performance, after cubist regression.

## Bayesian statistics in the assessment of the effects of COVID-19 drugs

Bayesian statistics has been recognized as a useful tool in developing new drugs and interpreting the results of clinical studies (11). One of the most important advantages of Bayesian statistics over the frequentist approach is its applicability in the analysis of data derived from trials using an adaptive trial design (12). This can be attributed to a fundamental difference between the two approaches – frequentist approach uses prior information only during the clinical study design and not in the results analysis, while Bayesian statistics allows combining and complementing newly obtained information with prior information in each step of the research: design, conduct, and data analysis (11). Several studies are currently using Bayesian methods to investigate the efficacy of potential COVID-19 drugs. One of them is “Hydroxychloroquine versus Azithromycin for Hospitalized Patients with Suspected or Confirmed COVID-19 (HAHPS)” (13). For data analysis during the course of the trial and after its end, the authors proposed the use of a hybrid Bayesian-frequentist approach for interim monitoring to allow rapid, contextual assessment of the available evidence. Another study is that by Griffiths et al (14), entitled “AGILE-ACCORD: ACcelerating COVID-19 dRug Development-Phase I / II trial platform,” which aims to test several COVID-19 drug-candidates. Bayesian inference will be used to determine the efficacy of the doses via a randomized Bayesian group-sequential trial with a time-to-event primary endpoint. In addition to analyzing the results of clinical trials, Bayesian computational approaches have also been used to identify potential drugs against SARS-CoV-2. Ekins et al (15) propose the application of *in silico* methods to accelerate SARS-CoV-2 drug discovery. Computer methods that could be used for these purposes are molecular docking for drug repurposing and machine learning methods to produce predictive models that can then be used to screen compound libraries and suggest new compounds to test. As an example, the authors developed several models for ACE2, SARS-CoV, and MERS-CoV based on ECFP6 descriptors and a Bayesian algorithm. Moreover, Angeletti et al (16) used fast-unconstrained Bayesian approximation to investigate the presence of mutations in ORF1ab SARS-CoV-2 region caused by selective pressure on the virus. A mutation discovered in the endosome-associated-protein-like domain of the nsp2 protein could explain why this virus is more contagious than SARS.

## Bayesian statistics in disease diagnosis

Given the rapid spread of SARS-CoV-2 virus and its effects on the respiratory system, it is critically important to develop diagnostic methods that are faster than reverse transcription polymerase chain reaction (17). Several studies have proposed the development of diagnostic methods based on image classification using deep learning. Namely, COVID-19 has been shown to cause abnormalities in the lung tissue visible on the chest x-rays and CT images as ground-glass opacities. However, in some cases, these changes are not easily distinguishable from those caused by other diseases, which is why the backbone of the system are the experts who could interpret the images. Ucar and Korkmaz (18) introduced a system called COVIDiagnosis-Net. Given the imbalance problem of the public data set used for ANN training, to optimize the hyper-parameters of the model, the authors used an offline well-defined augmentation process and Bayes-SqueezeNet. Their model outperformed the COVID-Net (19) trained on the same data set using non-Bayesian optimization methods, while reaching a test accuracy of 0.983. COVIDiagnosis-Net enabled the classification of chest x-rays into Covid, Normal, and Pneumonia classes, with an accuracy of above 96% for all classes. Similar results were obtained by Bahadur Chandra et al (20), who developed a system based on a two-phase classification approach (normal vs abnormal and nCOVID-19 vs pneumonia) using a majority vote based classifier ensemble of five benchmark supervised classification algorithms. During the learning process, all the optimizable learning hyper-parameters were tuned using the Bayesian automatic optimization method.

## COVID-19 data in R statistical program environment

**Figure 1:**
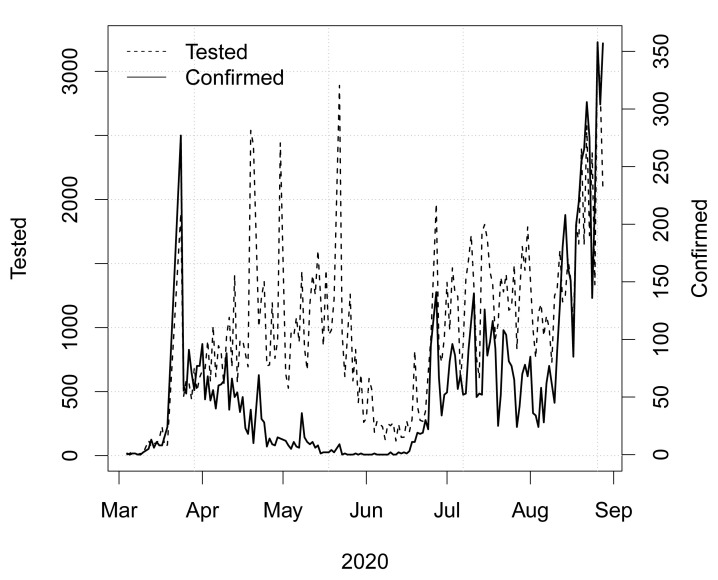
The number of tested persons and coronavirus 2019-confirmed cases in Croatia from March to the end of August 2020. The data were obtained from Johns Hopkins University Center for Systems Science and Engineering using the R package “*COVID19*.”

**Figure 2:**
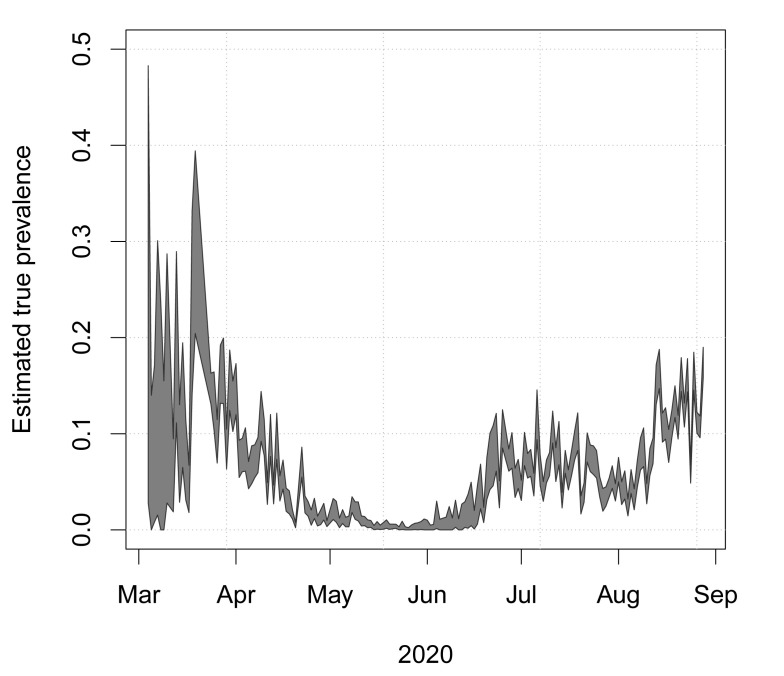
The estimated highest true prevalence range of coronavirus 2019 in Croatia according to the daily data obtained from Johns Hopkins University Center for Systems Science and Engineering using the R package “*COVID19.*” The true prevalence was estimated from the apparent prevalence in a Bayesian framework using the package “*prevalence*.”

Within the R statistical environment, three packages currently provide access to data on the COVID-19 pandemic: “*COVID19*,” “*coronavirus*,” and “*COVID19.Analytics.”* The “*COVID19*” package provides seamless integration with World Bank Open Data, Google Mobility Reports, and Apple Mobility Reports (21). The “*coronavirus”* package provides access to daily data by country from the Johns Hopkins University Center for Systems Science and Engineering (JHU-CCSE) databases (22). The “*COVID19.analytics*” package gives access to live worldwide JHU-CCSE data on COVID-19 and provides basic analysis tools and functions to investigate these data sets (23). Figure 1 shows the data for Croatia obtained directly from the JHU-CCSE using “*COVID19*” package, while Figure 2 shows the true prevalence based on daily data. Although the number of confirmed cases has increased (Figure 1), by the time of writing this column the range of the highest expected estimated true prevalence was still smaller than it was at the beginning of the epidemic. Thus, with the strictest conditions given in the statistical model (ie, sensitivity = 1 and specificity = 1), the highest expected true prevalence in Croatia still does not exceed 20%. If we take this in consideration, people at increased risk, ie, people with a higher probability to test positive (those who have been in contact with infected persons or have symptoms) in Croatia are still mostly tested. Therefore, it can be expected that the true prevalence is significantly lower (one order of magnitude smaller) than the most anticipated calculated one.
